# Comparing the Effectiveness of Polymer Debriding Devices Using a Porcine Wound Biofilm Model

**DOI:** 10.1089/wound.2015.0683

**Published:** 2016-11-01

**Authors:** Holly N. Wilkinson, Andrew J. McBain, Christian Stephenson, Matthew J. Hardman

**Affiliations:** ^1^ Faculty of Life Sciences, The Healing Foundation Center, The University of Manchester, United Kingdom.; ^2^ Faculty of Medical and Human Sciences, Manchester Pharmacy School, The University of Manchester, United Kingdom.; ^3^ Crawford Healthcare Limited, Knutsford, United Kingdom.

**Keywords:** wound biofilm, debridement, *Pseudomonas aeruginosa*, *Staphylococcus aureus*

## Abstract

**Objective:** Debridement to remove necrotic and/or infected tissue and promote active healing remains a cornerstone of contemporary chronic wound management. While there has been a recent shift toward less invasive polymer-based debriding devices, their efficacy requires rigorous evaluation.

**Approach:** This study was designed to directly compare monofilament debriding devices to traditional gauze using a wounded porcine skin biofilm model with standardized application parameters. Biofilm removal was determined using a surface viability assay, bacterial counts, histological assessment, and scanning electron microscopy (SEM).

**Results:** Quantitative analysis revealed that monofilament debriding devices outperformed the standard gauze, resulting in up to 100-fold greater reduction in bacterial counts. Interestingly, histological and morphological analyses suggested that debridement not only removed bacteria, but also differentially disrupted the bacterially-derived extracellular polymeric substance. Finally, SEM of post-debridement monofilaments showed structural changes in attached bacteria, implying a negative impact on viability.

**Innovation:** This is the first study to combine controlled and defined debridement application with a biologically relevant *ex vivo* biofilm model to directly compare monofilament debriding devices.

**Conclusion:** These data support the use of monofilament debriding devices for the removal of established wound biofilms and suggest variable efficacy towards biofilms composed of different species of bacteria.

**Figure:**
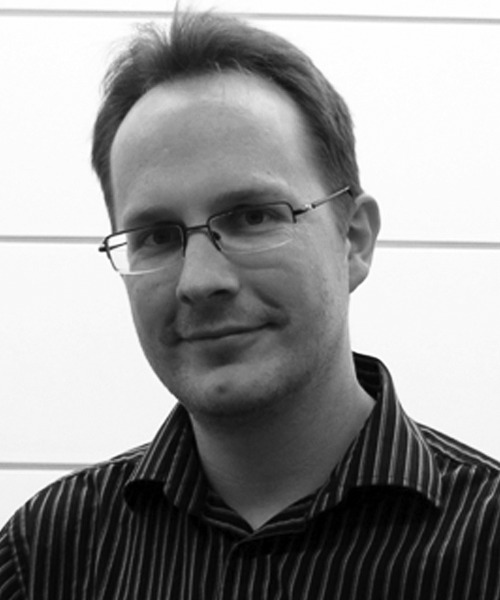
**Matthew J. Hardman, PhD**

**Figure:**
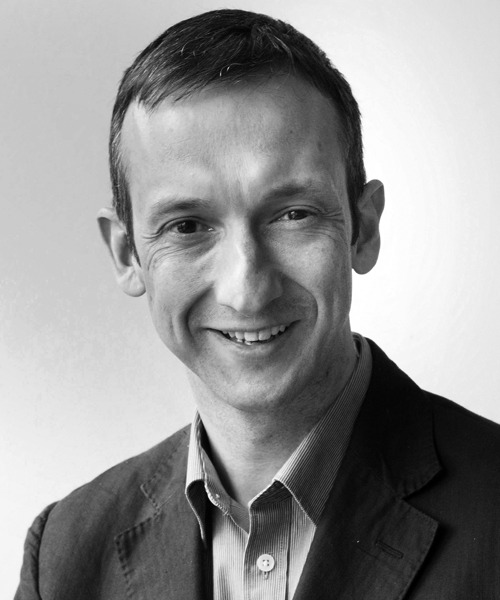
**Andrew J. McBain, PhD**

## Introduction

Nonhealing chronic wounds remain a major area of unmet clinical need, leading to increased patient morbidity and mortality, while imposing a significant financial burden on healthcare providers worldwide.^1,2^ Wound chronicity can arise through malfunction at any stage of repair and can be influenced by local (*e.g.*, ischemia) and systemic factors (*e.g.*, age, malnutrition, and disease), along with imbalances in cytokine levels and growth factors.^1^ One of the most discussed extrinsic causes of chronicity is the presence of infection, where opportunistic “critical” colonization of a wound by microorganisms can lead to the formation of a biofilm.^3–5^ Biofilms are becoming increasingly implicated in pathological healing due to their resistance to the host immune defence and transient responses to most therapeutic treatments.^5,6^ Established biofilms have a number of distinguishing characteristics that contribute to their resistant phenotype, most notably physiological and physiochemical effects resulting from encapsulation in a tough protective layer of extracellular polymeric substances (EPS).^7^

While current wound management involves a multidisciplinary team approach, debridement to remove necrotic, devitalized, and infected tissue remains a primary consideration in wound management.^8^ Historically, debridement has been suggested to promote healing by removing nonviable ischemic tissue and exposing underlying vascularized tissue, triggering endogenous reparative processes.^9^ More recently, it has been suggested that debridement may also promote healing through the physical disruption and removal of established wound biofilms.^7^

## Clinical Problem Addressed

Traditional debridement techniques, including sharp excision and curettage can be invasive and painful, requiring professional administration.^10^ A number of alternative, less costly debridement modalities have been developed over recent years, such as autolytic and mechanical debridement. Classical mechanical debridement involves the removal of necrotic tissue by scrubbing and stripping with cotton gauze.^11^ However, scrubbing and stripping can be invasive, painful, and may scrub the wound of valuable repair cells, depending on wound etiology and nature.^12^ Notably, the efficacy of simple cotton gauze at removing wound biofilms remains largely untested.^13^ Recently, new, mechanical, polymer fiber debriding devices have emerged, designed to remove both established wound biofilms and devitalized tissue with minimal discomfort (*e.g.*, Debrisoft^®^; Activa Healthcare, Debrimitt^™^; Crawford Healthcare Ltd.^14^).

In this study, we report a detailed side-by-side comparison to test the efficacy of monofilament debriding devices versus traditional gauze material in mechanical debridement of established *ex vivo* porcine wound biofilms.

## Materials and Methods

### Growth of biofilms

Single-colony inoculates of *Pseudomonas aeruginosa* (NCTC10781) and *Staphylococcus aureus* (NCTC13277) were grown overnight in Mueller Hinton (MH; Oxoid) broth, at 37°C with 220 rpm shaking (Innova 44; New Brunswick Scientific). The (O/N) cultures were diluted to obtain densities at c. 10^7^ colony forming units per milliliter (CFU/mL), verified by spread plating and colony counts. Sterile filter membranes (0.45 μm thick; Merck Millipore Ltd.) were inoculated with 10 μL of diluted cultures and incubated on MH agar plates for 48 h at 37°C (as in Merritt *et al.*^15^). The resultant 48 h established biofilms were added to wounded porcine skin.

### Porcine skin preparation

Porcine skin was collected of schedule 1 killed female pigs (weighing 43–45 kg) from the abattoir, epilated using dry razors and stored at −80°C until use. Defrosted skin was cut into 1.3 × 1.3 cm^2^ and wounded by completely removing the epidermis (No. 22 blades; Swann Norton). The skin squares were sequentially washed in sterile phosphate buffered saline containing 5 ug/L TWEEN 20 (PBST), 0.6% sodium hypochlorite (in PBST), and 70% ethanol (in PBST). Finally, tissue was washed thrice in sterile phosphate-buffered saline (PBS) to remove solvent residues (modified from Yang *et al.*^16^). To maintain a humid environment for bacterial growth, skin squares were placed in six-well plates on sterile PBS-soaked absorbent pads. A 48-h established biofilm was added to the wounded surface of each porcine biopsy, ensuring even coverage. Nonbiofilm controls were prepared in parallel. Biopsies were incubated at 37°C for an additional 24 h to allow further biofilm maturation and attachment to the wounded porcine skin.

### Mechanical debridement and sample collection

**Figure 1.**
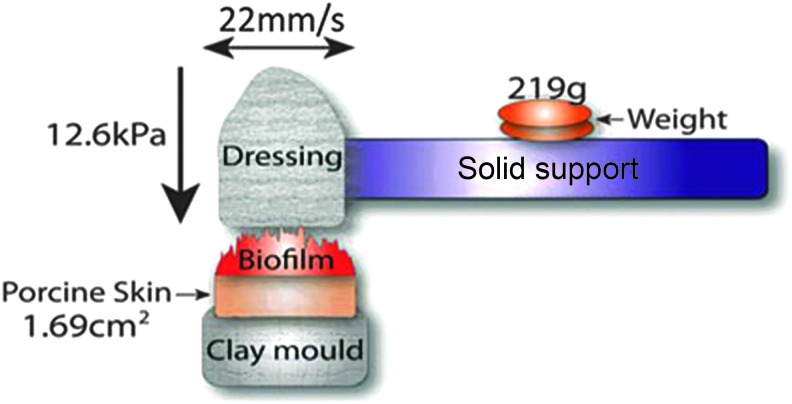
Debriding apparatus. Samples were debrided using an SD Mechatronik ZM-3 toothbrush simulator (Germany). Diagram of the debriding experiment setup. Debriding devices (gauze, Debrisoft^®^, or Debrimitt™) were attached to solid supports and inserted into the simulator. Porcine skin biofilms were attached to custom fabricated clay moulds positioned directly beneath the debriding devices. A weight of 219 g was placed at the center of each brush, exerting a force of 12.6 kPa per sample. Debridement consisted of 50 linear strokes at a speed of 22 mm/s.

Debriding devices (gauze, Debrisoft^®^, and Debrimitt^™^) were cut into strips, firmly stapled to toothbrush heads, and loaded into an SD Mechatronik ZM-3 tooth brushing simulator (Fig. 1A). The porcine biofilm biopsies were glued onto custom fabricated clay moulds and mounted in the toothbrush simulator. Each sample was brushed in a linear manner for 50 cycles at 22 mm per second, with a travel length of 10 mm and a constant force of 12.6 kPa (Fig. 1B). Separate samples were collected for PrestoBlue^™^ cell viability analysis (*n* = 3), bacterial viability counts (*n* = 3), and scanning electron microscope (SEM) preparation (*n* = 2) and embedded in optimum cutting temperature (OCT) solution for histological analysis (*n* = 2). In addition, *S. aureus* and *P. aeruginosa* 48-h membrane biofilms (before addition to porcine skin) were also collected to confirm the presence of established biofilms (data not shown).

### PrestoBlue^™^ *in situ* bacterial viability assay

Following debridement, the PrestoBlue^™^ Cell Viability Reagent (Invitrogen, Life Technologies) was applied evenly to the full surface of each tissue square and incubated for 3 min. Images were captured (Nikon D3200 Digital SLR camera with a Nikon AF-S DX Micro NIKKOR 40 mm f/2.8G Lens) and percentage cell viability calculated using ImagePro-Plus software (Media Cybernetics, Inc., Rockville, MD, USA).

### Bacterial viability counts

Each sample was cut into small (<1 mm) pieces using an aseptic technique and sterile scalpel blades (No. 22 blades; Swann Norton), then vortexed in 1 mL MH broth with 5 mL sterile glass beads (Borosilicate, 3 mm diameter; Sigma-Aldrich) for 30 s. The resultant resuspended bacteria were serial diluted and plated in triplicate to determine viable CFUs per biofilm.

### Scanning electron microscopy

Tissue samples (∼2 mm diameter), cut into trapezium shapes to identify orientation, were fixed for 4 h in 2% glutaraldehyde/paraformaldehyde in HEPES buffer, followed by transfer through an ETOH gradient (10%, 25%, 50%, 70%, 90%, and 3 × 100% for 15 min each). Critical point drying was performed using an E3000 (Quorum Technologies) in 100% ETOH. Samples underwent three exchanges of ETOH for liquid CO_2_ before heating and critical point drying. Samples were mounted onto stubs, placed in an argon vacuum, and sputter coated (SC7690; Quorum Technologies) with gold (Quanta Feg 250; FEI Company). Images were taken at three points per sample at a range of magnifications using a high-vacuum SEM. Gauze, Debrisoft^®^, and Debrimitt^™^ were also collected post-treatment, processed and dried for SEM as above, and imaged using low-vacuum SEM.

### Histological analysis of frozen tissue sections

OCT embedded samples were cryosectioned at 12 μm (CM3050 S; Leica Biosystems) and stored at −80°C until use. Before staining, sectioned slides at −80°C were brought to −20°C and fixed with cold acetone for 2 min, which was then left to evaporate at room temperature (<20 min). Slides were immersed in PBS to remove any residual OCT. Modified Gram–Twort staining was achieved following Bancroft and Gamble,^17^ DPX mounted and imaged using an Eclipse E600 microscope (Nikon) and SPOT camera (Image Solutions, Inc.). Biofilm thickness was quantified from Gram–Twort images using ImageJ software (NIH). Concanavalin A (ConA) staining for EPS (Alexa Fluor^®^ 488 conjugate, Molecular Probes^™^; Thermo Fisher Scientific) was performed O/N at 4°C. Sections were mounted in VECTASHIELD^®^ with 4′,6-diamidino-2-phenylindole (DAPI; Vector Laboratories Ltd.) to counterstain bacterial DNA. Similarly, sections were stained with Acridine Orange (Sigma-Aldrich) to visualize bacteria on host tissue. Fluorescent *in situ* hybridization (FISH), using PNA FISH^®^ kits (AdvanDx, Inc.) with *P. aeruginosa* and *S. aureus* probes, was used to show species specificity. Sections were imaged on an Olympus Snapshot fluorescence microscope using fluorescein isothiocyanate, DAPI, and TEXAS RED filters. *S. aureus* and *P. aeruginosa* membrane biofilms were embedded in OCT, sectioned, and then stained as above.

### Statistical analyses

Statistical tests were performed on all *S. aureus* and *P. aeruginosa* quantitative data (PrestoBlue^™^ cell viability, viability counts, and biofilm thickness), where treatment (uninoculated skin, established “control” biofilms, gauze dressing, Debrisoft^®^, and Debrimitt^™^) was plotted against viability/abundance/thickness. For viability counts and PrestoBlue^™^ viability data, one-way analysis of variances (ANOVAs) were performed in R v3.2.2 (R Development Core Team) with accompanying Tukey *post hoc* analyses. Nonpaired *T* tests were performed for biofilm thickness data.

## Results

### Comparative quantification of biofilm removal postdebridement

Biofilms of *P. aeruginosa* and *S. aureus* established on wounded porcine skin were subjected to controlled mechanical debridement with defined speed, duration, and pressure (Fig. 1). The biofilm remaining postdebridement was assessed by the following methods:

#### Surface area coverage of viable biofilm

**Figure 2.**
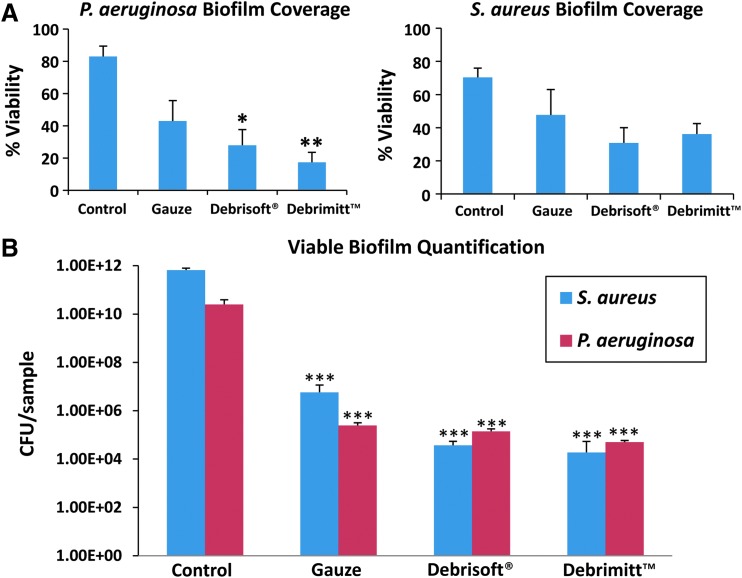
Assessment of debridement efficacy. **(A)** Quantification of biofilm coverage using a resazurin-based cell viability stain. In each case, debridement reduced biofilm coverage which achieved statistical significance for Debrisoft and Debrimitt application to *P. aeruginosa* biofilms. **(B)** Viable counting of bacteria remaining indicates statistically significant reductions in colony forming units following each treatment. Monofilament debridement outperformed traditional gauze. Graphs show means; error bars represent SEM. *p < 0.05; **p < 0.01; ***p < 0.001.

PrestoBlue^™^ was used to directly visualize the surface area of porcine skin with the remaining viable biofilm versus untreated established biofilms (Fig. 2A; ANOVA: *F*_3,11_ = 8, *p* = 0.004). In this study, Debrimitt^™^ and Debrisoft^®^ significantly reduced viable *P. aeruginosa* biofilm surface coverage compared to control biofilms that underwent no debridement (Debrimitt^™^, 95% confidence interval [CI 23.15–108.13], *p* = 0.003; Debrisoft^®^, 95% CI [12.45–97.43, *p* = 0.01). Of note, no significant difference was found between the gauze dressing and control *P. aeruginosa* biofilms (95% CI [−2.49–82.49], *p* = 0.067). A similar trend of surface area debridement efficacy was observed with *S. aureus* biofilms, although the data failed to reach significance (ANOVA: *F*_3,8_ = 3.16, *p* = 0.086).

#### Quantification of biofilm bacteria

Resuspending biofilm bacteria to gain quantitative viability data (Fig. 2B) revealed significantly reduced viable *P. aeruginosa* following debridement (ANOVA: *F*_3,20_ = 67.43, *p* < 0.001). Debrimitt^™^ treatment appeared most effective, resulting in a 6 log_10_ reduction (*p* < 0.001) versus control biofilms. Debrisoft^®^ and gauze debridement both led to 5 log_10_ reductions in viable *P. aeruginosa* (*p* < 0.001 and *p* < 0.001, respectively). *S. aureus* viability was also significantly reduced following debridement (Fig. 2B; ANOVA: *F*_3,11_ = 81.91, *p* < 0.001). In this study, both Debrimitt^™^ and Debrisoft^®^ treatment led to a 7 log_10_ reduction in *S. aureus* compared to control biofilms (*p* < 0.001). By contrast, treatment with a gauze dressing gave only a 5 log_10_ reduction in *S. aureus* (*p* < 0.001). Overall, the data suggest that all three debridement modalities remove viable biofilms of *S. aureus* and *P. aeruginosa* from porcine wound tissue, with Debrimitt^™^ proving most effective.

### Visualizing established biofilms *in situ*

#### Gram–Twort stain

**Figure 3.**
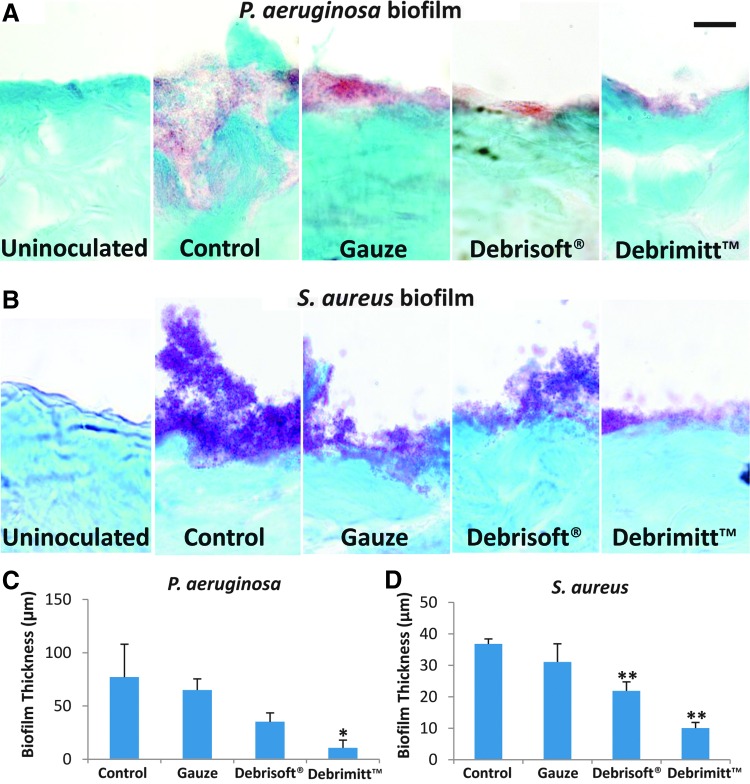
High-power modified Gram–Twort stained images confirm the extent of biofilm removal. **(A)** *P. aeruginosa* biofilm (*red*) was evident at the wounded surface in all but the untreated skin. All three debriding treatments reduced biofilm, with the most efficient removal following Debrimitt^™^ treatment. **(B)** *S. aureus* biofilm (*purple*) was also clearly attached to the surface of the wounded porcine skin. Again, debridement treatment substantially reduced the surface biofilm. Note, bacteria were observed not just at the surface but also integrated into the host tissue. **(C)** *P. aeruginosa* biofilm thickness was significantly reduced with Debrimitt debridement. **(D)** *S. aureus* biofilm thickness was significantly reduced with monofilament debridement. Date shows mean ± standard error of the mean. *p < 0.05; **p < 0.01. Bar **(A)** = 14.4 μm.

Frozen histological sections subjected to Gram–Twort staining confirmed both the presence and identity (*i.e.*, gram positive vs. gram negative) of *P. aeruginosa* (pink stain) and *S. aureus* (purple stain) biofilms on the porcine wound surface (Fig. 3). Control biofilms of gram-negative *P. aeruginosa* appeared as a pink continuous mass in the wounded porcine skin, mirroring the staining found in the *P. aeruginosa* membrane biofilms (data not shown). Following debridement, the amount (thickness) of visible bacteria decreased, with greatest reduction in visible biofilm in the Debrimitt^™^-treated group (*T*_9_ = 2.28, *p* < 0.05; Fig. 3C). Note, staining was absent from nonbiofilm, untreated porcine skin. *S. aureus*, a gram-positive bacterium stained purple with the Gram–Twort stain, was readily apparent on the surface of each control porcine wound biofilm and mimicked the staining of *S. aureus* membrane biofilms (data not shown). Thick aggregated clumps of *S. aureus* were readily visualized in nondebrided established biofilm tissue. These clumps of bacteria were greatly diminished following debridement, with the greatest reduction in biofilm thickness following monofilament debridement (Debrisoft^®^ and Debrimitt^™^, *p* < 0.01; Fig. 3D).

#### ConA, Acridine Orange, and FISH staining

**Figure 4.**
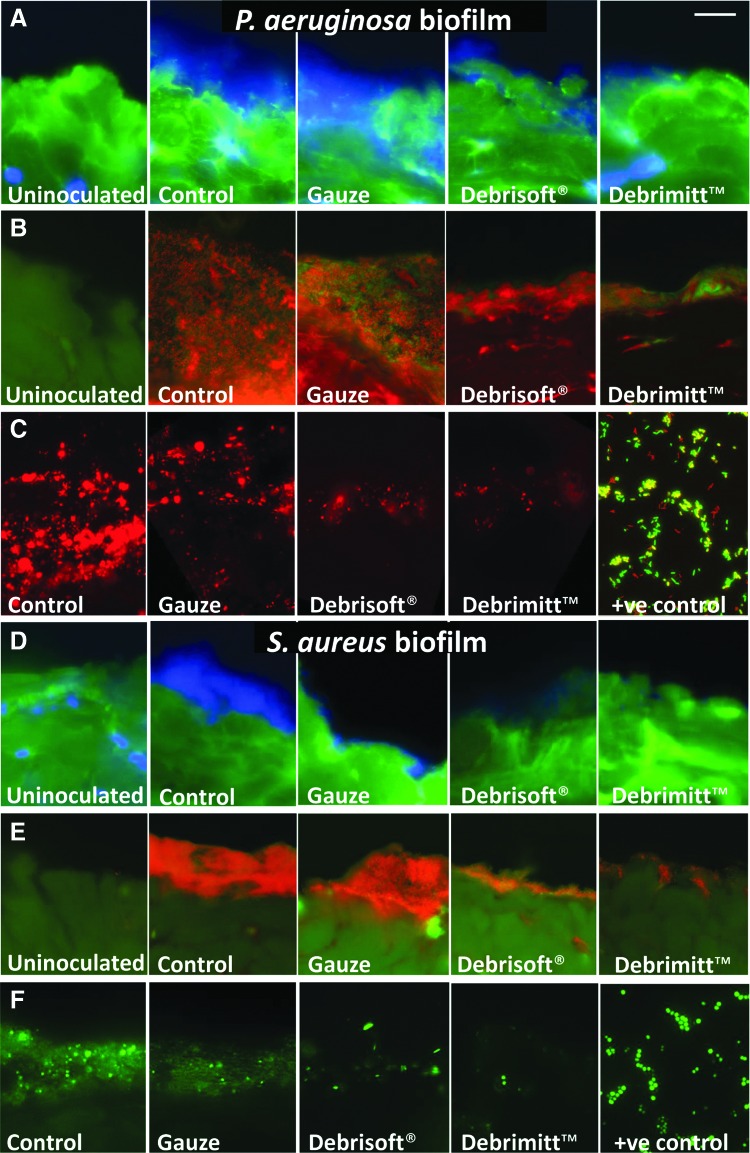
ConA, Acridine Orange, and FISH stained images confirm the extent of biofilm removal. **(A)** *P. aeruginosa* biofilms stain for bacterial EPS (*green*; ConA) with prominent DAPI (*blue*) bacteria visible at the porcine wound surface. **(B)** Acridine Orange staining for bacteria (*red*/*orange*) at the surface layers of the porcine wounds, with *green* host tissue. **(C)** FISH of *P. aeruginosa* (*red*) from the treatments, including a positive (+ve) slide containing *P. aeruginosa* (*red*), *Escherichia coli* (*green*), and *Klebsiella pneumoniae* (*yellow*). Debridement reduces apparent biofilm mass with Debrisoft^®^ and Debrimitt^™^ showing the most effective removal. **(D)** *S. aureus* biofilms exhibit DAPI stained bacterial mass (*blue*). **(E)** Acridine Orange staining for bacteria (*red*/*orange*) at the wound surface. **(F)** FISH of *S. aureus* (*green*) from the treatments, including a positive (+ve) slide containing *S. aureus* (*green*). *S. aureus* bacteria are prominent in nondebrided control samples, which substantially reduced following debridement, particularly following monofilament debridement treatments (Debrisoft^®^ and Debrimitt^™^). Bar **(A)** = 12.7 μm. ConA, concanavalin A; DAPI, 4′,6-diamidino-2-phenylindole; EPS, extracellular polymeric substances; FISH, fluorescent *in situ* hybridization.

Next biofilms were stained with a combination of ConA and DAPI counterstain (Fig. 4). Labeled lectins, such as fluor-conjugated ConA, which interacts with carbohydrates, are often used to indicate biofilm formation.^18^ However, EPS staining can vary due to biofilm-specific variation in polymeric substances.^19^ The bacteria within the biofilm mass could be visualized by DAPI (blue) (Fig. 4A, D), Acridine Orange (Fig. 4B, E), and FISH staining (Fig. 4C, F). Control *P. aeruginosa* and *S. aureus* biofilms were both prominent across the surface of the porcine wound. In keeping with the Gram–Twort staining, less biofilm was observed following each debridement treatment, with greatest reduction in the Debrimitt^™^ treatment group. Note, no biofilm mass (blue DAPI staining, orange/red Acridine Orange staining, or FISH staining) was observed in any of the uninoculated porcine skin samples (Fig. 4).

#### Scanning electron microscopy

**Figure 5.**
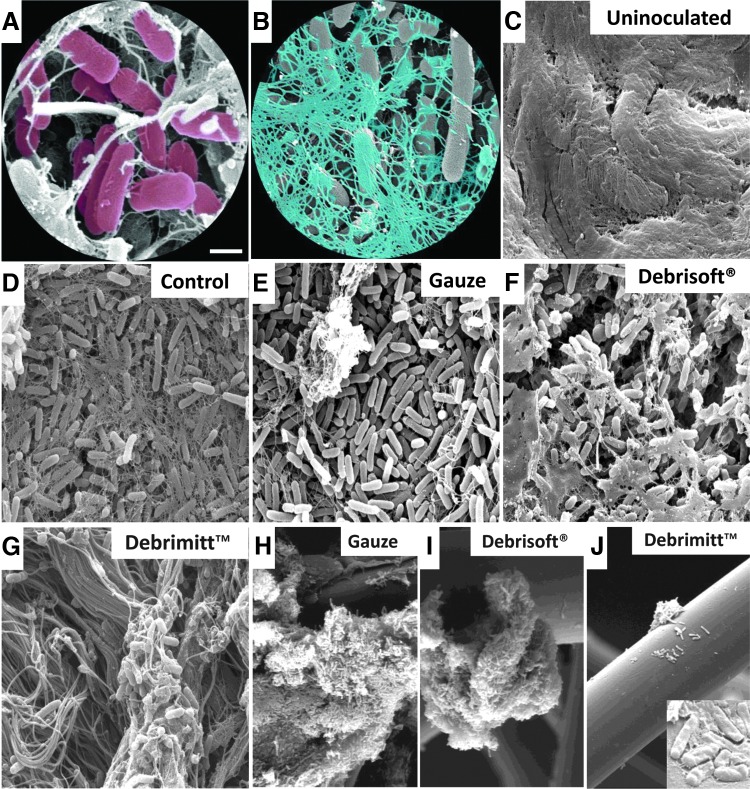
SEM images confirm *P. aeruginosa* biofilm removal following debridement. **(A)** Pseudocolored rod-shaped *P. aeruginosa* (*pink*). **(B)** Pseudocolored stringy EPS (*blue*). **(C)** Uninoculated porcine wound. **(D)** Control. **(E)** Gauze debrided. **(F)** Debrisoft^®^ debrided. **(G)** Debrimitt^™^ debrided. **(H–J)** Direct imaging of postdebridement devices reveal attached clumps of bacteria, with altered morphology [*inset*, **(J)**]. Bar **(A)** = 1 μm **(A**, **B)**, 5 μm (**C–G**, **J**; *inset*), and 20 μm **(H–J)**. SEM, scanning electron microscopy.

Finally, samples were subjected to SEM to directly visualize bacteria/tissue interaction. Control *P. aeruginosa* biofilm-treated skin (Fig. 5D) was covered with abundant rod-shaped *P. aeruginosa* (pseudocolored pink; Fig. 5A), encased in a stringy dehydrated EPS (pseudocolored blue; Fig. 5B), indicative of biofilm formation. As expected, uninoculated porcine wound samples lacked a visible bacterial biofilm (Fig. 5C). Interestingly, the surface of gauze debrided samples retained virtually full coverage of bacteria, but exhibited less EPS. A substantial proportion of the bacteria and EPS were removed by both Debrisoft^®^ and Debrimitt^™^ treatments, with remaining bacteria mainly residing within the porcine wound tissue (Fig. 5F, G). SEM imaging of the gauze, Debrisoft^®^, and Debrimitt^™^ following application revealed bacterial removal in large clumps, which remain on the fibers of the devices (Fig. 5H, J). Moreover, at high resolution, the *P. aeruginosa* bacteria retained on the Debrisoft^®^ and Debrimitt^™^ fibers appeared deformed and compressed compared to the *P. aeruginosa* removed by the gauze (Fig. 5J, inset). This suggests that debridement using Debrisoft^®^ or Debrimitt^™^ may adversely affect bacterial viability.

**Figure 6.**
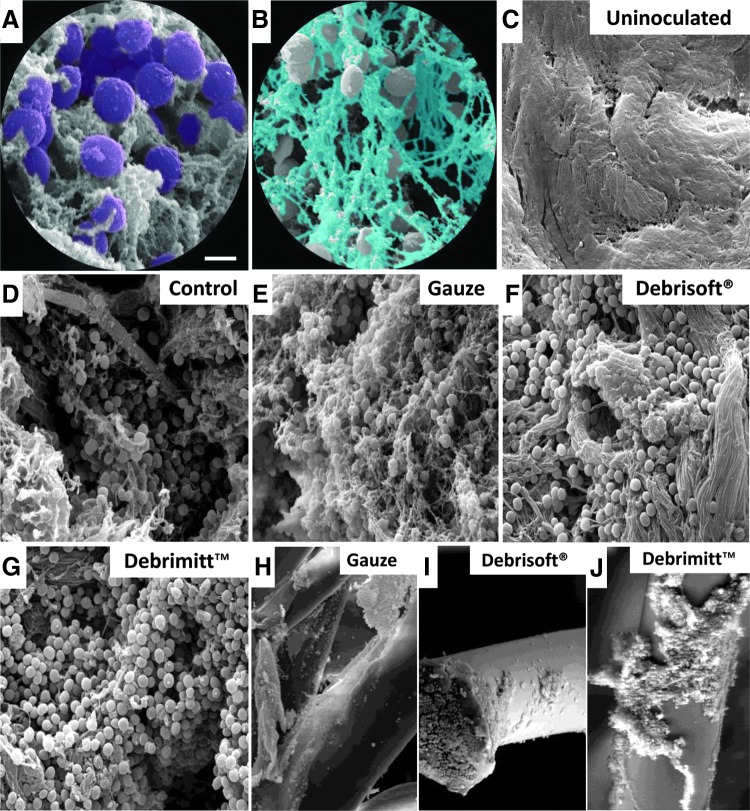
SEM images confirm *S. aureus* biofilm removal following debridement. **(A)** Pseudocolored coccoid-shaped *S. aureus* (*purple*). **(B)** Pseudocolored globular EPS (*blue*). **(C)** Uninoculated porcine wound. **(D)** Control. **(E)** Gauze debrided. **(F)** Debrisoft^®^ debrided. **(G)** Debrimitt^™^ debrided. **(H–J)** Direct imaging of postdebridement devices reveals attached clumps of bacteria. Bar **(A)** = 1 μm **(A**, **B)**, 5 μm **(C–G)**, and 20 μm **(H–J)**.

Similarly, *S. aureus* (pseudocolored purple, Fig. 6A) formed an extensive covering on the surface of control biofilm-treated porcine wounds (Fig. 6D), this time encased in a more globular EPS (pseudocolored blue; Fig. 6B). As with *P. aeruginosa*, *S. aureus* porcine biofilm skin subjected to gauze debridement retained many *S. aureus* bacteria with prominent EPS remaining (Fig. 6E). By contrast, Debrisoft^®^ and Debrimitt^™^ debridement appeared to remove the majority of surface EPS (Fig. 6F, G). Again, *S. aureus* bacteria were clearly visible on the monofilament device fibers following treatment (Fig. 6H, J). Interestingly, *S. aureus* appeared to be removed in large EPS encapsulated clumps, particularly evident following debridement with Debrimitt^™^.

## Discussion

Chronic wounds, often refractory to current treatments, remain a huge challenge in the clinic.^3^ Bacterial infection has long been implicated in impaired wound repair, where some studies suggest chronicity is prolonged by established bacterial biofilms comprising pathogens such as *P. aeruginosa* and *S. aureus.*^3,20,21^ Simple yet efficient removal of wound biofilms should therefore be particularly beneficial to promote wound repair. In this study, we have established an *ex vivo* single-species porcine biofilm model using *P. aeruginosa* and *S. aureus*. Using this model, and carefully controlled application parameters, we show that new polymer debriding devices are more effective at removing established wound biofilms than standard gauze. Indeed, combination of quantitative and morphological assessments revealed that the polymer debriding device Debrimitt^™^ was most effective at removing established biofilms from porcine wound tissue.

While virulence is associated with biofilm prevalence, bacterial loads of greater than 10^5^ bacteria per gram have been linked to nonhealing pathophysiology.^22^ Four log_10_ is also a key measure for bacterial reduction, being the FDA threshold criteria for dressing/device antibacterial efficacy claims. Indeed, it has been suggested that a 5 log_10_ reduction in wound bacteria would require 17 generations to recover, equating to around 12 h in a wound environment.^23^ Therefore, utilizing monofilament debriding products may open a time-dependent therapeutic window for cotreatment and biofilm eradication.^24^ In this study, biofilm viability plate spread counts indicate that monofilament debridement reduced wound biofilm bacterial loads by up to 7 log_10_ (against *S. aureus* biofilms), equating to over 16 h recovery time (based on *in vitro* planktonic culture growth rates).

A major strength of this study is the use of *ex vivo* porcine skin for biofilm establishment. Tissue studies have been indicated as more adequate than *in vitro* models for testing the significance of clinical treatments^4^ as they provide the opportunity for bacteria to interact with host tissues (reviewed in Roberts *et al.*^25^). The *ex vivo* porcine wound model used here provides a close physiological comparison to human wound tissue^26^ using clinically relevant bacteria.^21^ Indeed, SEM analysis reveals that in our biofilm model, bacteria are closely integrated into the porcine tissue, depositing EPS that form an interface with the host dermal fibers. *In vivo* models will provide a further level of relevance, allowing the contribution of host factors such as ischemia and necrosis on biofilm to be assessed.^27^ However, as animal studies are expensive, time consuming, and technically demanding, we suggest that *ex vivo* tissue models allow easier control of experimental variables and assessment of outcomes.^16^

By virtue of their protective EPS matrix, bacteria within complex biofilms benefit from increased resistance to treatments and host immunity versus planktonic species.^28^ The mechanism of EPS function has begun to be investigated. For example, inhibiting synthesis of the EPS component, exopolysaccharide alginate, in *P. aeruginosa* biofilms potentiated phagocytosis by activated human leukocytes.^29^ Further studies inhibiting EPS demonstrate reduced antibiotic resistance^28^ or complete inability to form biofilms (reviewed in Flemming and Wingender^30^). Interestingly, virulent biofilm-forming *P. aeruginosa* is known to synthesize exogenous polysaccharides for encapsulation and annealment to host tissues through carbohydrate-modulating genes such as algC^31^. Modulations in genes coding for motility and aggregation have been demonstrated in biofilm-forming *S. aureus* (reviewed in Jefferson^32^). Gene expression profiling of planktonic bacteria compared to biofilms has identified multiple genes which contribute to the sessile, antibiotic-resistant nature of biofilms.^33,34^

Despite these studies, identification of bacterial biofilms in wound tissue remains an area of contention.^35^ In this study, we used 48 h *ex vivo* established biofilms of *P. aeruginosa* and *S. aureus* based on previous published data.^4,16^ *In vitro* studies indicate *P. aeruginosa* biofilm growth within 10 h^22^ and certainly by 24 h.^36^ Similarly, *S. aureus*, known to establish more slowly than *P. aeruginosa*, formed biofilms in 24 h when grown on MH substrate.^37^ Although the patterns of EPS expression and the distinct polymers produced differ between species,^38^ SEM imaging of mature biofilms has previously depicted EPS as long strings (*P. aeruginosa*^30^) or cloud-like clumps (*S. aureus*,^4^) surrounding the bacteria within a biofilm. In this study, we demonstrate the same morphological features in our 48 h established *ex vivo* porcine skin biofilms.

A second important characteristic of established biofilms is the formation of microcolonies (shown by SEM^37^). Maintaining close proximity allows bacteria in biofilm microcolonies to exchange genetic information and make use of chemical signaling (quorum sensing^19^) to increase virulence.^39^ Our SEM data reveal that in the *ex vivo* porcine model, both *P. aeruginosa* and *S. aureus* establish EPS encapsulated microcolonies that are characteristic of established biofilms.^4^ Interestingly, our data agree with previous SEM studies suggesting that monofilament polymer devices structurally integrate wound debris.^40^ In the current study, we observed clumps of aggregated *P. aeruginosa* in all three test groups, while clumped aggregates of *S. aureus* were visible only on the monofilament devices (Fig. 6). Finally, our inclusion of uninoculated porcine skin for SEM analysis confirmed that wounded skin had been adequately cleaned to remove endogenous bacteria before establishment of species-specific biofilms. When combined with Gram–Twort, Con A, Acridine Orange, and FISH staining, to indicate the regional extent of biofilm presence and removal, our data indicate (i) established biofilm presence before debridement and (ii) improved efficacy of monofilament debridement versus standard gauze.

In summary, we describe a 48-h *ex vivo* porcine skin biofilm model that exhibits many features of the established biofilm phenotypes, including EPS production. Using this model, we demonstrate the efficacy of monofilament debridement as a clear alternative to more invasive debridement techniques currently used in the clinic. Quantitative assessment revealed the monofilament debriding device, Debrimitt^™^, to provide the greatest reduction in biofilm bacterial load. Intriguingly, our data suggest that monofilament devices effectively remove both bacteria and EPS and that the mechanical debridement process may damage bacteria *in situ*, further influencing viability. Our study highlights further opportunities to carefully and reproducibly test the efficacy of mechanical debridement *in vivo* and in clinical contexts.

## Innovation

This is we believe, the first study to directly compare the efficacy of monofilament debriding devices for the removal of established biofilm from skin. We report standardized conditions for the application and testing of debriding devices and a portfolio of assessments to quantitatively and qualitatively monitor biofilm removal. Finally, our data reveal differential efficacy toward defined bacterial species.

Key Findings

• Monofilament debridement efficiently removes established bacterial biofilms *ex vivo*.
• *S. aureus* and *P. aeruginosa* biofilms are differentially susceptible to debridement.
• Debridement removes both EPS and wound bacteria.

## About the Authors

**Holly N. Wilkinson, MRes,** is studying for a PhD in the Healing Foundation Centre at The University of Manchester**. Andrew J. McBain, PhD,** is a Professor of Microbiology at the University of Manchester. He leads a research group that focuses on the interaction of microorganisms with the human body in health and disease, and on clinical and environmental biofilms. **Christian Stephenson, BSc,** has a background in Chemistry with extensive experience in medical devices product development and wound management. **Matthew J. Hardman, PhD,** formerly the Edmond de Rothschild Senior Fellow in Ageing Research, is currently a senior lecturer in the Healing Foundation Centre at The University of Manchester. He received his PhD in Skin Developmental Biology under the supervision of Professor Carolyn Byrne at The University of Manchester. The primary focus of his research is elucidating molecular and cellular correlates to pathological wound healing.

## Abbreviations and Acronyms

ANOVA: analysis of variance
CFU: colony forming units
ConA: concanavalin A
DAPI: 4′,6-diamidino-2-phenylindole
EPS: extracellular polymeric substances
FISH: fluorescent *in situ* hybridization
MH: Mueller Hinton
O/N: overnight
OCT: optimum cutting temperature
PBS: phosphate-buffered saline
SEM: scanning electron microscopy
